# A WEb-Accessible comprehensiVE platform for automatic vestibular schwannoma segmentation and longitudinal volumetric tracking

**DOI:** 10.1093/noajnl/vdag138

**Published:** 2026-05-23

**Authors:** Riya Prashad, Gregory Szalkowski, Jen-Yeu Wang, Fred C Lam, Ahed H Kattaa, Vivek Sanker, Cynthia Chuang, Lei Wang, Lianli Liu, Qingying Wang, Yinheng Zhu, Mingli Chen, Iris Gibbs, Scott G Soltys, Erqi L Pollom, Elham Rahimy, David J Park, Yusuke S Hori, Steven D Chang, Hao Jiang, Weiguo Lu, Xuejun Gu

**Affiliations:** Department of Radiation Oncology, Stanford University, Stanford, California, USA; Department of Radiation Oncology, Stanford University, Stanford, California, USA; Department of Radiation Oncology, Stanford University, Stanford, California, USA; Department of Neurosurgery, Stanford University, Stanford, California, USA; Department of Neurosurgery, Stanford University, Stanford, California, USA; Department of Neurosurgery, Stanford University, Stanford, California, USA; Department of Radiation Oncology, Stanford University, Stanford, California, USA; Department of Radiation Oncology, Stanford University, Stanford, California, USA; Department of Radiation Oncology, Stanford University, Stanford, California, USA; Department of Radiation Oncology, The University of Texas Southwestern Medical Center, Dallas, Texas, USA; Department of Radiation Oncology, The University of Texas Southwestern Medical Center, Dallas, Texas, USA; Department of Radiation Oncology, The University of Texas Southwestern Medical Center, Dallas, Texas, USA; Department of Radiation Oncology, Stanford University, Stanford, California, USA; Department of Radiation Oncology, Stanford University, Stanford, California, USA; Department of Radiation Oncology, Stanford University, Stanford, California, USA; Department of Radiation Oncology, Stanford University, Stanford, California, USA; Department of Neurosurgery, Stanford University, Stanford, California, USA; Department of Neurosurgery, Stanford University, Stanford, California, USA; Department of Neurosurgery, Stanford University, Stanford, California, USA; Department of Radiation Oncology, The University of Texas Southwestern Medical Center, Dallas, Texas, USA; Department of Radiation Oncology, The University of Texas Southwestern Medical Center, Dallas, Texas, USA; Department of Radiation Oncology, Stanford University, Stanford, California, USA; Department of Radiation Oncology, The University of Texas Southwestern Medical Center, Dallas, Texas, USA

**Keywords:** auto-segmentation, longitudinal volume tracking, stereotactic radiosurgery, treatment follow-up, vestibular schwannoma

## Abstract

**Background:**

Vestibular schwannomas (VS) require long-term tracking for treatment decisions and outcome assessment. This study aims to develop a WEb-Accessible comprehensiVE (WEAVE) platform that combines AI-driven segmentation with a user-friendly interface to enable longitudinal volume tracking for disease assessment, planning, and monitoring.

**Methods:**

WEAVE was built using nnU-Net as the backbone for auto-segmentation, with 3 models trained and validated on combinations of various image modalities. Auto-segmentation performance was evaluated with multiple metrics including absolute and relative volume differences (AVD/RVD), Dice score, mean surface-to-surface distance, and 95th percentile Hausdorff distance (HD95). The platform features a central database with DICOM-RT import/export capabilities, and its interface is built using Rust and WebAssembly.

**Results:**

Three models demonstrated comparable performance without significant differences with mean Dice scores, AVD, and RVD ranged from (0.89 to 0.90), (0.11 to 0.13 cc), and (10.70% to 13.44%), respectively. Mean surface-to-surface distance and HD95 values were consistently low (0.14-0.19 and 0.74-0.88 mm, respectively). Average inference time was ∼60 s per case. The platform successfully enabled longitudinal tumor volume tracking and provided flexible visualization options, including single and multiple image views and a graphical representation of volume changes over time.

**Conclusions:**

WEAVE is a comprehensive platform that combines automated segmentation with longitudinal tracking to support VS management. The AI models achieved Dice scores comparable to interobserver variability in manual contouring, indicating clinical adequacy. The tracking capability provides consistency in treatment planning and monitoring and opens the possibility to advance AI-driven segmentation and streamline workflows for other intracranial pathologies.

Key Points

- Accurate AI auto-segmentation and longitudinal volume tracking facilitate VS management.
- A web browser-based, user-friendly platform with integrated dataflow enables practical clinical use.

Importance of the Study

Currently, there is no widely accepted clinical tool for automatic segmentation and longitudinal tracking of vestibular schwannomas (VS). We developed a browser-based platform that performs AI-enabled VS auto-segmentation and volumetric tracking over time. The platform runs in standard web browsers without local installation and supports database access, image visualization, and contour editing via a web client. It offers DICOM import/export for interoperability with clinical databases and treatment planning systems and is compatible with multiple image modalities. Future enhancements include integrating dose-prediction and plan-optimization algorithms to enable a comprehensive stereotactic radiosurgery workflow.

Vestibular schwannoma (VS), otherwise known as acoustic schwannoma or acoustic neuroma, is a slow-growing, benign tumor that arises from the vestibulocochlear nerve.^1^^,^^2^ Ipsilateral sensorineural hearing loss occurs in more than 90% of cases; other presentations include tinnitus, dizziness or imbalance, ataxia, trigeminal nerve dysfunction, facial nerve palsy, and vertigo.^3-6^ Clinicians often use the Koos classification system to grade these tumors based on linear measurement and anatomical positioning, which helps guide management strategies, such as observation, stereotactic radiosurgery (SRS), and microsurgical resection.^1^^,^^6-8^ However, linear measurements are often limited by subjective line placement^9^; in contrast, volumetric analysis provides a more objective metric.^10^ For SRS specifically, accurate volumetric delineation of the target (or tumor, VS in this case)^11^ is essential to maximize the radiation dose to the target to ensure the tumor control while sparing the adjacent critical structures such as the cochlea and brainstem to minimize radiation toxicity. In current clinical practice, post-contrast T1-weighted (T1c) MRI^12^ scans are considered the gold standard for VS delineation due to their high sensitivity and specificity^13^^,^^14^ and they are complemented by T2-weighted (T2) MRI and CT scans for radiation treatment planning. Because SRS is tumoristatic rather than tumoricidal, long-term tumor volumetric analyses are crucial to differentiate pseudo-progression from true regrowth and to assess treatment-related complications.^1^^,^^7^^,^^13^^,^^15-18^ While precise and repeated volumetric measurements are imperative to determine treatment efficacy and guide clinical decisions, this process is labor-intensive and requires significant expertise from highly trained neuroradiologists.

Deep learning (DL)-based auto-segmentation techniques have been gaining popularity in recent years as a time-saving measure to help reduce clinician workload, with an estimated 30% reduction in segmentation time.^17^ Several groups have developed and published DL-based auto-segmentation algorithms for VS, achieving varying levels of success. A review on VS segmentation by Nernekli et al^19^ noted that nearly all studies from 2010 to 2024 investigating machine learning approaches to VS segmentation have employed a U-Net or U-Net-type model, highlighting the efficacy of U-Net architecture. Various groups have further refined U-Net models by incorporating spatial attention modules,^10^ multi-scale simultaneous feature map generation,^20^^,^^21^ and sequentially stacked models.^22^ While all these algorithms are promising for clinical use, they failed to address the need for long-term longitudinal follow-up. Recently, Jester et al^13^ developed a software tool for assessing the growth of VS, which achieved a mean Dice score of 0.88 with their DL auto-segmentation model and enabled monitoring of tumor growth by overlaying sequential images with coregistration. However, the DL auto-segmentation model was developed only with T1c MRI, restricting its applicability in cases of gadolinium intolerance or CT-required SRS planning. Similarly, Lee et al^5^ developed a dual-path convolution neural network for longitudinal imaging analysis of VS in Gamma Knife-based SRS and treatment follow-up using both T1c and T2 MR, yet their framework remains incompatible with CT. Furthermore, the applications developed by Jester et al^13^ and Lee et al^5^ are both standalone tools without an integrated dataflow to communicate with clinical software and/or a user-friendly interface to facilitate user interaction, which may have limited practicality in the clinical setting. Consequently, there remains a critical need for an ­accessible, multi-modal platform that streamlines auto-segmentation and volumetric tracking to support disease assessment, treatment planning, and posttreatment monitoring.

To address the unmet clinical needs and the limitations of existing methods, we propose a WEb-Accessible comprehensiVE (WEAVE) platform that assists VS disease assessment, SRS treatment planning, and posttreatment monitoring. The WEAVE platform consists of (1) a back-end software package, for database management, image registration, preprocessing, and segmentation, and (2) a front-end web interface for visualization and user interaction. The platform supports multiple imaging modalities, including both CT and MRI, ensuring its suitability for various radiation platforms. For instance, both Varian and Elekta linear accelerators (Linacs), as well as the Accuray CyberKnife, require a combination of CT and MRI images for planning, whereas Gamma Knife SRS planning relies solely on MRI images. In this project, we established multiple DL-based VS auto-segmentation models corresponding to different image modality combinations and incorporated them into the WEAVE platform. We further utilized AI-based auto-segmentation to assess VS volumes over time, enabling longitudinal tracking and visualization of VS volume changes on web browsers. We evaluated the performance of DL-based auto-segmentation with 20 diverse clinical cases from our institution, as well as 242 cases from a publicly available The Cancer Image Archive (TCIA) dataset. We validated the longitudinal tracking modules with 5 additional cases to demonstrate the clinical applicability of the WEAVE platform.

## Methods

### Platform Overview

**Figure 1.**
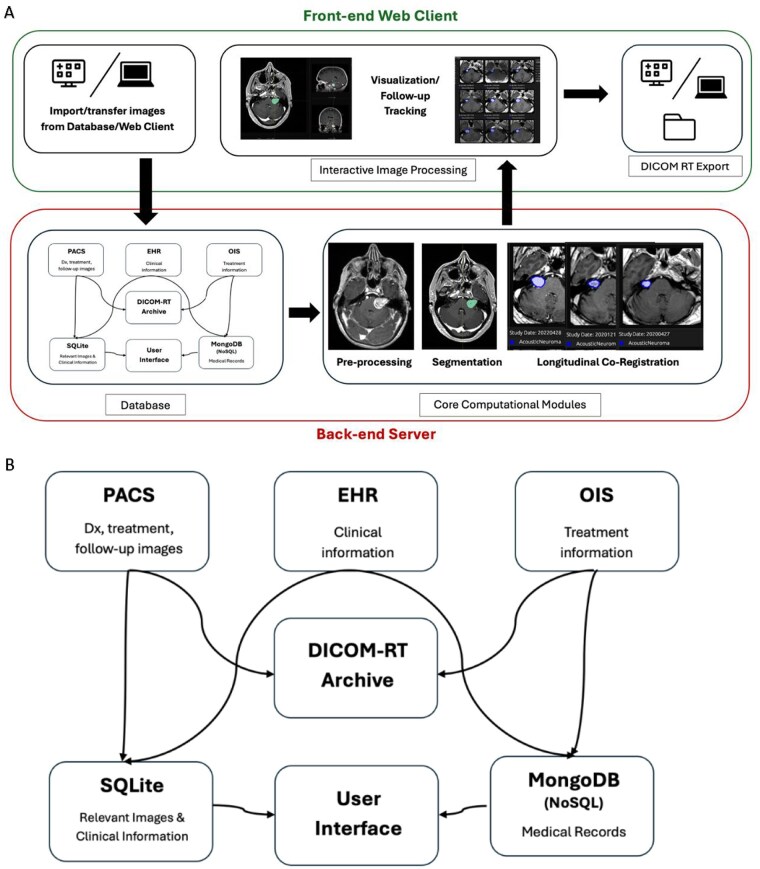
(A) The overall workflow of WEAVE platform; (B) schema of dataflow with database formed by SQLite, DICOM-RT Archive, and MongoDB. EHR, Electronic Health Record; OIS, Oncology Information System; PACS, Picture Archiving and Communication System; WEAVE, WEb-Accessible comprehensiVE.

Figure 1A illustrates the overview of the WEAVE platform, currently deployed in our own institution to assist clinical and research tasks. The WEAVE platform consists of a front-end web client and a back-end server. The web client is implemented in Rust compiled to WebAssembly (Wasm). Rust provides predictable, low-overhead performance, and fine control over data layout, with compile-time memory safety and no garbage collector. When targeted to Wasm, computationally intensive routines can achieve performance close to native in modern browsers, which is well suited to 3D image processing and visualization. Wasm’s contiguous, growable linear memory (backed by typed arrays) makes it straightforward to manage large image volumes and stream data efficiently. By leveraging Rust + Wasm, alongside GPU-accelerated rendering via WebGL/WebGPU, our client delivers interactive, near-real-time processing and visualization of large medical images for clinical workflows.

The WEAVE platform can be accessed via any common web browser. Within the institutional network, granted users can connect to the web client using the server’s IP/Port address. This web-based design allows users to utilize the developed tools without needing to install software on local computers. The web client facilitates user interactions such as database access, image visualization, and task selection. The back-end server is responsible for executing tasks received from the web client, such as data format conversion, database integration, VS segmentation, and image coregistration for VS volume tracking. As demonstrated in Figure 1A, the overall workflow of the WEAVE platform can be described as follows:

1. *Image receiving*: the platform receives images either through Picture Archiving and Communication System (PACS) transfer or by user import from the web client.
2. *Database integration and auto-image processing*: after receiving images, the backend server will integrate the data into the database, then automatically preprocess images, auto-segment VS, and coregister new images with previous ones if there are any.
3. *Visualization and interactive image processing*: the processed image will be displayed on the web client, and the user can modify and postprocess contours using the available tools.
4. *DICOM-RT export*: after interactive image processing, users can export the refined contours to the local drive or the designated DICOM-RT server (eg SRS treatment planning system [TPS]).

### Back-End Database

In the SRS clinic, data are often scattered across 3 systems: images (diagnosis, treatment, and follow-up) are stored in the PACS, clinical information in the Electronic Health Record system, and radiotherapy (RT) treatment information in the Oncology Information System). For the WEAVE platform, we constructed a basic centralized database (SQLite) within the hospital’s private cloud but limited the storage to relevant images and clinical information. We established a DICOM-RT archive to manage DICOM-RT data acquired along the entire RT care path, from diagnosis and planning to treatment and follow-up. The supported data types include Images (MRI, CT, etc.), Registration, RTStruct, RTDose, RTPlan, RTImage [Digitally Reconstructed Radiograph (DRR)s, portal images, etc.], and RT Record. We built a NoSQL database using MongoDB to support document, key-value, graph, and column-wide store, enabling storage of medical records containing diagnosis, treatment, and follow-up information in JSON-like documents. The user interface can fetch and query both image and nonimage data. Overall, the database stores RT patients in a digital format by integrating data from the various clinical data repositories, as illustrated in Figure 1B, serving as an effective and efficient “one-stop” source accessible via a web app.

### Backbone 3D nnU-Net Model for VS Auto-Segmentation

Our auto-segmentation DL models are built on nnU-Net,^23^ which possesses self-configuration that automatically determines optimal training hyperparameters based on a given dataset, eliminating the time and effort that domain-specific researchers previously needed to empirically find these settings. In addition, nnU-Net can handle large, high-resolution 3D images and can incorporate multiple imaging modalities. We trained and validated nnU-Net v2 with our institution data and integrated the trained models into the WEAVE platform.

### Dataset

We retrospectively reviewed patients with VS who underwent SRS in our institution between 2016 and 2025 for inclusion in the current study. Through the institutional IRB’s approval, we were able to build testing sets for the auto-segmentation models through chart review of eligible VS cases. Due to the retrospective nature of data collection, the requirement for informed consent was waived by the IRB. Postoperative cases and those lacking T1c and/or T2 MRI images were excluded, resulting in 113 patient cases forming the *Institutional Dataset*. For SRS treatment, patients undergo a CT scan, which is used for treatment planning in conjunction with the most recent MR images. Together, the CT, T1c, and T2 MRI images, as well as manually delineated contours (considered the gold standard), were extracted from our CyberKnife TPS to train and validate DL models. Images used in this study have stereotactic image quality, with high image resolution with in-plane measurements of ≤1.0 mm and slice thickness of ≤1.5 mm. To evaluate the generalizability of our trained model, we further collected the Vestibular-Schwannoma-SEG dataset provided by TCIA. This *TCIA dataset* consists of 242 cases with T1c and T2 imaging and corresponding contours, containing T1c images with resolutions of 0.4 × 0.4 × 1.0-1.5 mm and T2 images with resolutions of 0.5 × 0.5 × 1.0-1.5 mm.

### Models and Training

Considering different clinical application scenarios, we used the institutional data to train 3 nnU-Net models to accommodate different input image modalities: (1) VS_CT-T1-T2: using CT, T1c, and T2 MRI as inputs, suitable for CT-based SRS treatment planning on the platforms of CyberKnife and other general Linacs; (2) VS_T1-T2: using T1c and T2 MRI as inputs, applicable to MRI-only SRS as well as longitudinal growth monitoring and posttreatment follow-up; (3) VS_T1: using T1c MRI only as inputs, applicable to scenarios where patients may have T1c images only, which is particularly useful for studying historical data where only T1c MRI is available. The nnU-Net software was downloaded from the GitHub repository (https://github.com/marcuswirtz-snkeos/nnUNet) and compiled into standalone executables using PyInstaller (https://pyinstaller.org/en/stable/) with a Python environment on a workstation equipped with an NVIDIA RTX 6000 Ada Generation GPU and an Intel Xeon w5-2445 CPU. All training and inference processes were conducted using these precompiled executables, allowing the customized nnU-Net to function as an off-the-shelf application. For model training and inference, all images were resampled to the resolution of 1.0 × 1.0 × 1.0 mm^3^, and each imaging modality was treated as a separate input channel. The robust auto-configuration capabilities of nnU-Net simplify the model training process. Additionally, users are offered 3 options to tailor their model training according to their data availability, data format, and computational resources. With available 3D images and high-end GPU, we chose a 3D full-resolution configuration.

The institutional data were divided into training and testing in a ratio of 4:1, resulting in 93 cases for training and 20 for testing. The nnU-Net utilizes 5-fold cross-validation during training and automatically splits the training dataset further into training and validation cases for each fold, ending with 74-75 cases used for training and 18-19 for validation in each fold. The training of nnU-Net for VS auto-segmentation was terminated at 999 epochs with a converged loss score, requiring approximately 65 h in total. Right after the model interference, postprocessing, such as contour smoothing and island points removal, was applied. The average auto-segmentation time, including both model inference and postprocess time, was ∼60 s per case. To further test the impact of training sample size on models’ performance, we randomly selected 10, 30, 50, and 70 cases and trained 4 additional VS-T1 models. These 4 additional trained models were evaluated on both our institutional 20-sample testing dataset and the TCIA dataset.

### Evaluation Metrics

We evaluated the performance of the 3 models quantitatively using the following metrics: absolute volume difference (AVD) and relative volume difference (RVD), Dice score, mean surface-to-surface distance (MSSD), and Hausdorff distance (HD) in the 95th percentile (HD95). The manually contoured clinical volumes were used as ground truth and compared to the AI-segmented volumes in each case. AVD is the absolute volume difference between the ground truth contour and the AI-segmented one, and RVD is the percentage difference calculated by dividing AVD by the ground truth volume. The dice score calculates the volumetric similarity between the 2 structures, with a score of 1.0 representing perfect overlap and 0 representing no overlap. Mean surface-to-surface distance quantifies the average Euclidean distance from a voxel on the ground truth to the closest voxel on the AI-segmented volume. Euclidean distances are obtained via a 3D distance transform on the binary masks, with values scaled by the voxel spacing to yield millimeters. The distances are calculated in both directions and then averaged to provide the final value. A smaller MSSD signals a better segmentation. Hausdorff distance measures the maximum surface-to-surface discrepancy between the ground truth and AI segmentations: the largest overall surface voxels in 1 mask of the shortest Euclidean distance to the other mask. It is an indication of how well the segmentation boundaries align, and the 95th percentile (HD95) was used to reduce sensitivity to outliers. Hausdorff distance values closer to 0 indicate better agreement between the ground truth and AI-segmented volumes.

## Results

### Front-End Interface

#### Interface for a single image

**Figure 2.**
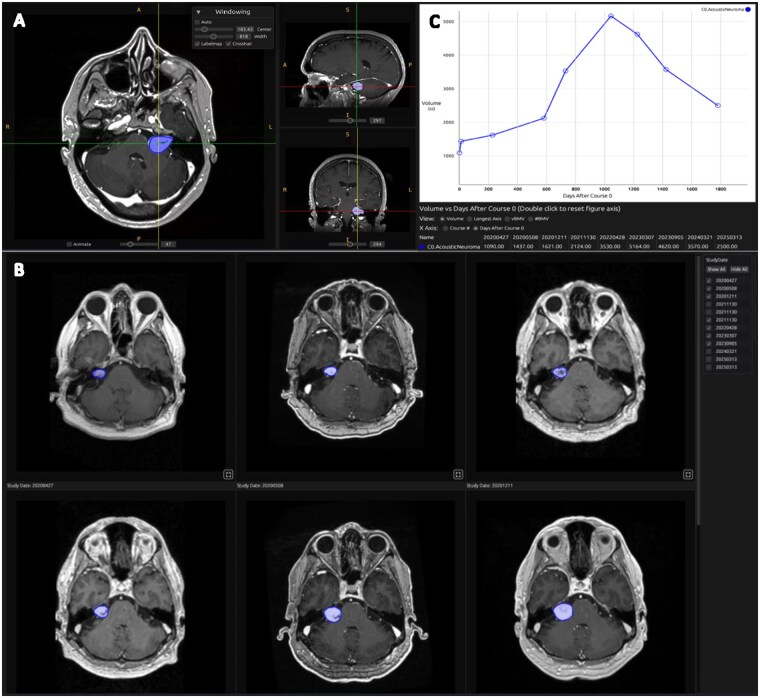
(A) Single image viewer displaying patient scan across axial, coronal, and sagittal planes with contours. Users can edit contours and complete other postprocessing tasks; (B) longitudinal tracking interface: multiple view with a checklist allowing users to choose which timepoints are displayed. Users can also choose individual timepoints to be highlighted; (C) graphical representation of volume change over time generated by the platform’s tracking capabilities.

The WEAVE landing page lists all imported patients. Selecting a patient opens the patient overview page, where all available assets are listed, including imaging series, DICOM-RT objects, and relevant documents. Choosing an image launches the single image viewer (Figure 2A), which displays the study in 3 orthogonal views (axial, coronal, and sagittal) alongside AI-segmented contours. Users can review and edit contours, switch among views and image series, and access postprocessing tools via the “Tasks” menu on the patient overview page. The “TrackVS” button will lead users to the tracking interface, which is detailed in the following section. The “Export” button generates DICOM-RT files for transfer to a designated server.

#### Interface for series of images and lesion tracking

There are various views available in the tracking interface; the “Single” view, where users can switch between timepoints, and the “Multiple” view (Figure 2B), where clinicians can view the tumor across timepoints and assess growth patterns. Both views contain a control center on the top right as well, allowing flexibility in visualization. A report can be generated as shown in Figure 2C, which graphically depicts the changes of tumor volume over time. This report can be customized to include/exclude certain timepoints and/or structures. Notably, the images can be automatically sent to the WEAVE database, as it is integrated with the clinical repositories during the initial system setup. Image loading during daily use would take 5-10 s, with a similar amount of time taken for the generation of volume tracking variables and graphs.

### Segmentation Accuracy

#### Quantitative results

The performance of the 3 models trained with 93 institutional cases was evaluated on the institutional testing dataset (20 cases) and the TCIA dataset (242 cases). Results were compiled into a set of summary statistics, as seen in Table 1. The mean values are shown with SD, as well as the median values, to lessen the impact of outliers. Overall, the trained models perform marginally better on the institutional testing dataset rather than on the TCIA dataset, with a mean Dice of 0.89 vs 0.86. Overall, the VS_CT-T1-T2 model had slightly better performance than the VS_T1-T2 and the VS_T1 models. All results were within close enough range of each other that there were no significant differences.

**Table 1.** Summary of the performace of the model trained with 93 institutional dataset across metrics on the institutional and TCIA testing datasets

Institutional dataset						
	Mean ± SD			Median		
Metrics	VS_CT-T1-T2	VS_T1-T2	VS_T1	VS_CT-T1-T2	VS_T1-T2	VS_T1
AVD (cc)	0.11 ± 0.27	0.13 ± 0.38	0.13 ± 0.52	0.04	0.06	0.04
RVD (%)	10.70 ± 9.12	11.23 ± 7.86	13.44 ± 0.12	8.42	7.07	10.45
Dice score	0.90 ± 0.05	0.89 ± 0.04	0.89 ± 0.05	0.91	0.90	0.89
MSSD (mm)	0.19 ± 0.26	0.14 ± 0.13	0.17 ± 0.24	0.09	0.10	0.11
HD95 (mm)	0.74 ± 0.57	0.76 ± 0.54	0.88 ± 0.95	0.56	0.57	0.57
TCIA dataset						
	Mean ± SD			Median		
Metrics	VS_CT-T1-T2	VS_T1-T2	VS_T1	VS_CT-T1-T2	VS_T1-T2	VS_T1
AVD (cc)	–	0.40 ± 0.91	0.34 ± 0.87	–	0.16	0.10
RVD (%)	–	0.17 ± 0.22	0.15 ± 0.24	–	0.11	0.07
Dice score	–	0.86 ± 0.21	0.86 ± 0.22	–	0.92	0.92
MSSD (mm)	–	0.85 ± 3.67	0.66 ± 3.61	–	0.08	0.08
HD95 (mm)	–	1.43 ± 5.14	0.96 ± 2.62	–	0.41	0.41

The TCIA dataset does not include the CTs so the VS_CT-T1-T2 model was not assessed here.

Abbreviations: AVD, absolute volume difference; HD95, Hausdorff distance in the 95th percentile; MSSD, mean surface-to-surface distance; RVD, relative volume difference.

When testing on our institutional dataset, AVD ranges from 0.11 to 0.13 cc, while RVD between the clinical manual and AI contours ranges from 10% to 13% across models. Note that the ground truth volumes of the testing cases range from 0.23 to 9.61 cc, where the small volume causes large RVD. The mean of MSSD is low across all, between 0.14 and 0.19 mm, while the HD95 is relatively high, ranging from 0.74 to 0.88 mm. The same trend follows for the SD, with HD95 having a larger SD than MSSD. Also, the VS_CT-T1-T2 model had slightly better performance than the VS_T1-T2 and the VS_T1 models, while all results were within close enough range of each other that there were no significant differences.

When testing models on the TCIA dataset, we observe that VS_T1 model resulted in an average Dice score of 0.86 ± 0.22 (0.92 median), with 71% (173/242) cases giving a Dice >0.90 and 88% of cases (214/242) giving a Dice >0.80. The VS_T1-T2 model performed similarly to the VS_T1 model, with a mean Dice score of 0.86 ± 0.20 (median 0.92), with 73% (175/242) of cases giving a Dice >0.9. Since the TCIA dataset did not provide the CT images for the included cases, we could not evaluate the VS_CT-T1-T2 model. According to the results from institutional data testing, we would expect that VS_CT-T1-T2 could have similar performance to VS-T1 and VS-T1-T2.

**Figure 3.**
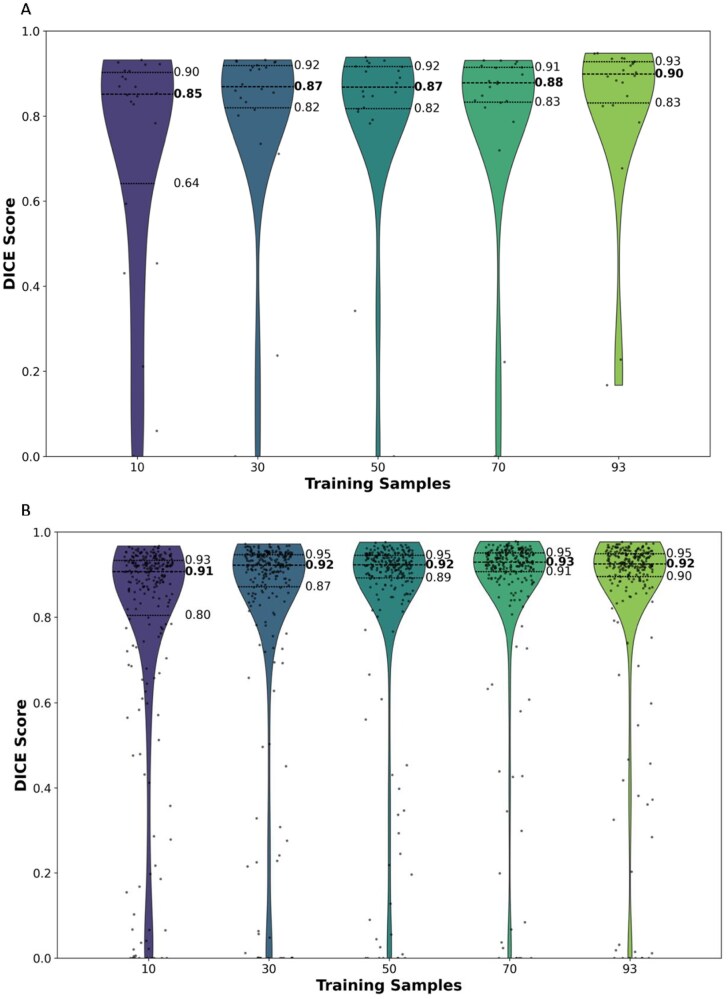
Violin plots displaying the testing results from models with varied training sample sizes, including 10, 30, 70, 70, and 93 samples. (A) Institutional testing dataset (*n* = 20): The results show a progressive increase in the median Dice score from 0.85 to 0.90. (B) TCIA dataset (*n* = 242): The median Dice score remains above 0.90 stabilizing at approximately 0.92 as the sample size increases. TCIA, The Cancer Image Archive.

We further analyzed the impact of the number of training cases on models’ performance by training an additional 4 VS-T1 models using *N* = 10, 30, 50, and 70 institutional data samples to supplement our original model (*N* = 93). These 5 models are tested on both the institutional testing dataset (*N* = 20) and the TCIA dataset (*N* = 242), and the testing results are illustrated in Figure 3. For the institutional dataset testing (Figure 3A), the median Dice score increased progressively from 0.85 at *N* = 10 to 0.90 at *N* = 93. The interquartile ranges initially decrease substantially between 10 and 30 samples and remain consistent after the training sample reaches 30. For the TCIA dataset testing (Figure 3B), the median Dice score remains above 0.90 even with only 10 training samples, stabilizing at approximately 0.92 as the sample size increases. These results show that a sample size >50 should be sufficient for training an accurate, stable model. Notably, the first and third quartiles for the 50, 70, and 93-sample models are nearly identical, indicating consistent performance after sample size >50. Another observation is that some cases in the TCIA dataset have almost a zero Dice score. We carefully examined those cases and found some have a lack of contrast uptake in the tumor, leading to poor segmentation, and others were in postsurgical status or present in nonstandard locations.

#### Qualitative analysis

**Figure 4.**
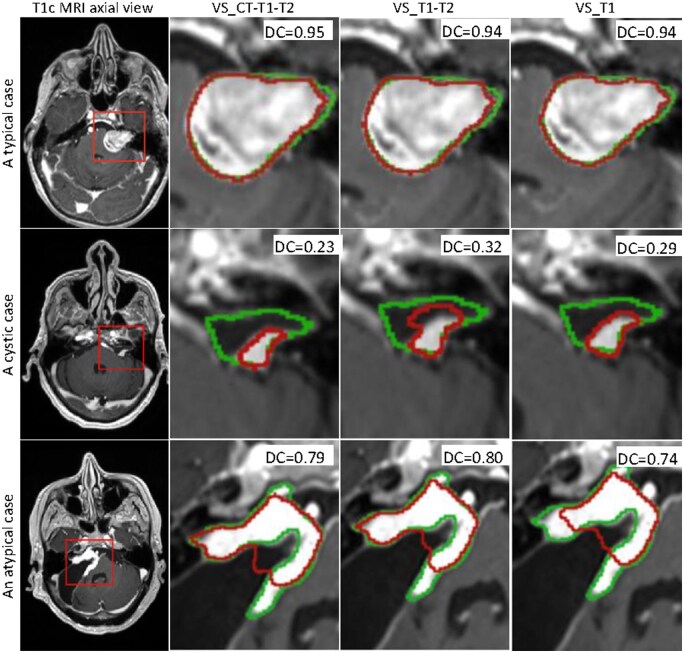
Qualitative illustration of sample auto-segmentation results on row I: a typical case; row II: a cystic case and an atypical case across different models using different models. Here, the ground truth contours are in green and AI contours in red; Dice Coefficient (DC) of each case is shown on top right of each sub-figure. T1C, post-contrast T1-weighted; VS, vestibular schwannomas.

Figure 4 shows segmentation results across the 3 models from several test cases, with the ground truth shown in green. Figure 4, row I, demonstrates a typical case, with Dice score between 0.94 and 0.95 across models. This test case is representative of the VS that is presented to our clinic for evaluation. The boundary lines of the tumor volume within the cerebellopontine angle of the posterior fossa are well defined but are less clear within the Internal Auditory Canal (IAC). Figure 4, row II, shows a case of a cystic VS with an enhancing solid component emerging from the IAC and a nonenhancing T1 hypointense fluid-filled cyst extending into the cerebellopontine angle. The Dice scores for this case range from 0.23 to 0.32, with the VS_T1T2 model performing the best. All models failed to contour the hypointense cystic portion of the schwannoma. Figure 4, row III, represents an irregularly shaped solid portion of a Koos Grade 4 vestibular schwannoma with a large cystic component causing mass effect on the brainstem that posed challenges for accurate AI segmentation. The Dice scores ranged from 0.74 to 0.80, but all 3 models were over-contoured and under-contoured on similar parts of the tumor.

### Cases Study With Longitudinal Follow-up

**Figure 5.**
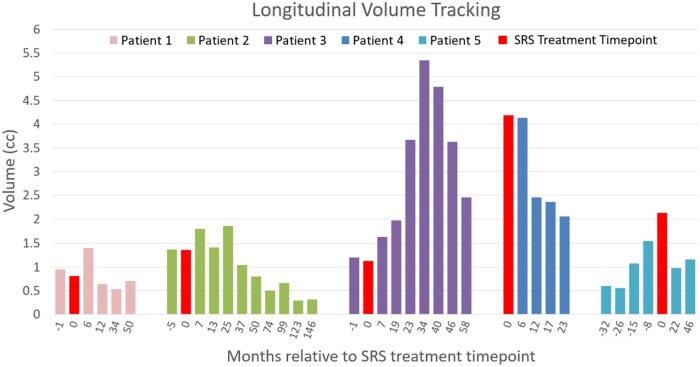
Longitudinal volume tracking of 5 patients: volume changes of tumor volumes are shown over time with time ranges presented in months relative to SRS treatment timepoint (red bar, month 0).

In this study, we randomly selected 5 cases and used WEAVE’s volumetric tracking to assess longitudinal tumor changes. As shown in Figure 5, in the early post-SRS period (0-7 months), volumes were either stable (patient 4) or showed transient increases (patients 1 and 2). At longer follow-up (≥12 months), most cases (1, 2, 4, 5) demonstrated volume decline, with patient-specific timing and magnitude. These patterns are consistent with expected post-SRS effects, transient enlargement due to treatment-related edema, microvascular changes, or cystic dynamics, followed by stabilization or shrinkage indicating tumor control. In contrast, case 3 exhibited marked enlargement after treatment. The auto-segmentation results were qualitatively verified by a neurosurgery fellow (5+ years of experience), and clinical notes corroborated interval enlargement (though without quantitative measurements), demonstrating concordance between WEAVE’s outputs and clinical documentation. Notably, these 5 patients’ surveillance MRIs before and after SRS were not acquired with stereotactic protocols; accordingly, we observed variability in the auto-segmentation model’s performance across protocols. Thus, protocol variability introduces uncertainty into volumetric tracking on the current WEAVE platform.

## Discussion

To the best of our knowledge, the WEAVE platform is a first-in-class, comprehensive, web-accessible platform for auto-segmentation and longitudinal monitoring of VS. The platform integrates image preprocessing and coregistration with an auto-contouring pipeline that produces an interactive visualization for clinician review and editing of contours, thereby facilitating a more efficient clinical workflow. The WEAVE platform supports coregistration of longitudinal imaging, enabling dependable, reproducible, and rapid tracking of tumor dynamics and volumetric change in a single online user interface and workspace. Given the generally predictable tumoristatic effects of SRS on the posttreatment growth kinetics of VS over the course of years, our platform allows for uploading of serial imaging modalities and facilitates integration with various SRS platforms, such as GammaKnife, CyberKnife, conventional Linacs, etc., thus broadening its clinical accessibility.

WEAVE’s user interface allows clinicians to visualize tumor volumes, edit contours as needed, and perform additional postprocessing tasks. Users can choose to work with a single image for treatment planning or with a series of longitudinal images for long-term monitoring. Through integration with clinical information systems, the platform can retrieve up-to-date patient data to support informed clinical decision-making. By automating preprocessing and segmentation, WEAVE allows clinicians to focus on care decisions while improving consistency and reducing inter-observer variation. Once contours are finalized, users can export contours to the clinical TPS, enabling flexible integration with existing clinical workflows. To our knowledge, no platforms currently provide this combination of auto-segmentation, longitudinal image coregistration/volume tracking, interactive editing, and seamless clinical integration for end-to-end VS management.

Segmentation models’ performance was evaluated against manual contours as the gold standard. To ensure high accuracy, contours from our institutional dataset were produced via a two-step expert process: initial delineation by a neurosurgeon followed by review and revision by an attending radiation oncologist. Across segmentation models (VS_T1, VS_T1-T2, and VS_CT-T1-T2), performance was broadly comparable, with no significant differences for most pairwise comparisons. Furthermore, consistent performance of our model on both the institutional and TCIA datasets demonstrated strong generalizability. Given the cubic-centimeter scale of VS targets, AVD and RVD between AI-generated and clinical contours were small, and Dice scores are high around 0.9. For small targets, Dice is particularly sensitive because even minor deviations constitute a relatively large fraction of the total volume. Moreover, multiple studies have documented inter-observer variability in manual contouring.^24-26^ As a result, further performance gains may be constrained when training on ground-truth delineations generated by multiple physicians, as in our dataset. Notably, all models achieved mean Dice scores of ∼0.9, approaching the typical range of human-to-human agreement.^27^ However, lower performance in atypical cases underscores the necessity of expert oversight. We therefore recommend that this AI tool serve as a clinical adjunct for decision support rather than a replacement for expert expertise.

In longitudinal use, coregistration and standardized visualization within WEAVE enabled consistent volumetric trend analysis across time, supporting reliable assessment of tumor dynamics within the same clinical workspace. Clinicians can view individual scans, or multiple timepoints at once, to allow them flexibility in studying and assessing tumor changes over time. This is essential for tracking how well the tumor responds to SRS, as well as to determine the course of treatment initially. The generation of a graph representing longitudinal volumetric change is beneficial for clinicians, allowing them to quantify the changes in addition to visualizing them. As depicted by Figure 5, the platform supports cohort analysis across diverse case types, guiding treatment selection and tracking post-SRS tumor response.

Despite WEAVE’s advantages, several limitations remain. First, segmentation accuracy decreases in certain phenotypes. In Figure 4 (row II), a cystic VS shows that although the T1c + T2 (VS_T1T2) model better identifies the cystic component, overall performance across models is suboptimal, likely due to a few representative cystic cases^28^ in our training dataset. In Figure 4 (row III), an irregularly shaped tumor leads to underperformance for all models, again reflecting limited exposure to such morphologies. Second, performance varies across imaging protocols. Because the models were trained on high-resolution stereotactic MRI, accuracy can decline on lower-resolution surveillance MRIs, as we observed in longitudinal tracking. To address these limitations, we will expand the training dataset to include a broader range of imaging resolutions and protocols to improve the model’s robustness and generalizability. Also, for cases underrepresented in our institutional clinical practice, we will pursue multi-institutional collaborations to further diversify and scale the dataset. Third, as this longitudinal study was limited to 5 demonstration cases, future research utilizing a larger dataset is necessary to validate its clinical utility. Future work will also extend WEAVE’s treatment-planning capabilities with dose prediction and automated plan generation, enabling clinicians to import images, auto-generate contours, and produce an initial, dose-optimized plan with organs-at-risk sparing, streamlining end-to-end planning.

## Data Availability

The data used in this manuscript is available on reasonable request and subject to institutional approval.
